# Assessment of the Effect of Meaningful Occupations on Motivation by Orbitofrontal Cortex Activation Using Near-Infrared Spectroscopy

**DOI:** 10.7759/cureus.66541

**Published:** 2024-08-09

**Authors:** Shintaro Ishikawa, Keisuke Fujii, Kenta Kunoh, Daisuke Kimura

**Affiliations:** 1 Department of Rehabilitation, Yamada Hospital, Gifu, JPN; 2 Department of Rehabilitation Occupational Therapy Course, Faculty of Health Science, Suzuka University of Medical Science, Suzuka, JPN; 3 Department of Occupational Therapy, Faculty of Medical Sciences, Nagoya Women’s University, Nagoya, JPN

**Keywords:** motivation, ofc, nirs, occupation, occupational therapy

## Abstract

Background: Meaningful occupations are those perceived as important by an individual. Research on meaningful occupations relies on subjective data and requires qualitative inquiries. Therefore, assessing the meaning of occupations using objective methods is challenging. As orbitofrontal cortex (OFC) activation is part of the reward system network involved in motivation, it could aid in assessing the meaning of occupations.

Objective: We aimed to investigate the effect of meaningful occupations on motivation by measuring OFC activation using near-infrared spectroscopy (NIRS).

Methods: Eight young and healthy participants were enrolled in this study. The occupation was set as “cooking,” and its importance was confirmed using the Canadian Occupational Performance Measure (COPM). NIRS was performed using an OEG-16 (Spectareteh Inc.). The target task involved watching a cooking video, while the control task consisted of looking at a “+” sign on a blank sheet of paper. OFC activation was measured based on changes in oxygenated hemoglobin (oxy-Hb) concentration using a block design. Participants with COPM scores of eight or more were classified into the “meaningful occupation performance” group, while those with scores of seven or lower were classified into the “meaningful occupation non-performance” group. Changes in oxy-Hb concentrations between the two groups were compared using the Mann-Whitney U test.

Results: Four participants were assigned to the meaningful occupation group (frequency of implementation: various times per week for all participants), and four participants were assigned to the meaningful occupation non-performance group (frequency of implementation: various times per week for one participant, various times per month for one participant, and various times per year for two participants). Statistical analysis revealed significant differences in the changes in the oxy-Hb concentration in the left and bilateral OFC.

Conclusion: This study suggests that it is important to focus on meaningful occupations that individuals consider important in order to activate the reward system and increase motivation.

## Introduction

Occupations involve activities people engage in during their lives, encompassing subjective experiences and personal meaning [1]. Occupational therapy is a clinical technique that focuses on meaningful occupations and aims to improve physical and mental health and occupational performance by enabling people to participate in meaningful activities in their daily lives [2]. Therefore, occupational therapy plays an important role in shaping identity through individuals’ subjective experiences [3].

An occupation is an activity that people do to fill their time and give meaning to their lives through their daily lives [4]. A meaningful occupation is an activity an individual considers important in life [5].

When choosing an occupation, an individual’s feelings, identity, and lifestyle play a crucial role; thus, it is challenging to assess the meaning of an occupation in an individual’s life by using objective indicators. Research on the meaning of occupations relies on subjective data, such as surveys of personal experiences. Qualitative studies, such as interviews, are necessary to determine the characteristics of an occupation. However, to scientifically determine the effectiveness of occupational therapy interventions, given the strengths and weaknesses of subjective assessment methods, the question arises as to whether it is possible to determine the meaning of an occupation using objective methods or whether these methods should be supplemented [6]. Furthermore, from a neurophysiological perspective, the motivation and decision-making involved in active participation in an occupation are explained in terms of the neural basis thought to exist in the brain, highlighting the need for evaluation from this neurophysiological perspective [7]. The meaning of an occupation is closely related to an individual’s motivation to perform that occupation in their own life [8]. Additionally, the reward system network, which includes the orbitofrontal cortex (OFC), is thought to be involved in the motivation related to the meaning of an occupation [9]. OFC has long been associated with updating stimulus-reward associations and with encoding the current value of rewards. One of the most important factors in decision-making is estimating the value of the available options. Subregions of the prefrontal cortex, including OFC, have been considered essential for this process. Effective connectivity, in which the activity in one part of the network influences the activity in another part, has been proposed as a means of elucidating these networks [10]. OFC activity can be measured using near-infrared spectroscopy (NIRS) [11]. Therefore, we hypothesized that by measuring OFC activation using NIRS during interventions that focus on occupations meaningful to individuals, we could understand the activity of the entire reward network and determine whether occupational interventions focusing on meaningful occupations have an impact on the individual’s motivation.

In this study, we aimed to measure OFC activation using NIRS and to investigate the impact of focusing on meaningful occupations on motivation.

## Materials and methods

Participants

The study included eight healthy individuals (six men and two women, mean age 23.6±1.4 years). Inclusion criteria were individuals between 22 and 25 years of age who reported good health, had received a full explanation of the purpose and methods of the study, and had provided their consent to participate. For health status, we defined it as good if the participant had undergone a medical examination at their place of work, the results showed no abnormalities, and their health was considered good. Individuals with physical or mental illness and those who had participated in another clinical trial within the previous six months were excluded. The study was approved by the research ethics committee, of which the first author is a member (Approval No. 2022-211).

Experimental environment

The researcher and participant sat on chairs facing each other at a 90° angle, and task instructions were given while they were seated. The environment was designed to control attention by blocking visual information using partitions around the area. Additionally, participants were instructed to maintain their posture and keep their limbs still during the task. These settings eliminated other confounding factors, such as eye movement, posture, and upper and lower limb movements.

Occupation setting

In previous research, meaningful activities were defined as "activities that individuals consider important in their daily lives" [5]. In this study, the "meaning" of meaningful occupation was operationally defined as "important to the individual". In addition, the occupation was set as “cooking.” The reason for selecting “cooking” was that this is a broad concept that encompasses everything from the enjoyment of cooking to the act of cooking as a job [12], facilitating that participants would add personal meaning to the context of the occupation. In addition, occupational therapy has used cooking as an intervention in various situations throughout its history. Cooking is a familiar task in everyday life that involves psychosocial processes and has the potential to have a positive impact on health [13]. The frequency and importance of cooking as an occupation were assessed using the Canadian Occupational Performance Measure (COPM). The COPM is an occupational therapy assessment scale developed by the Canadian Occupational Therapy Association in 1990. It measures changes in the way a person or family member views occupational performance for a variety of disabilities and individuals [14]. The COPM is an outcome measure designed to be used by occupational therapists to assess client outcomes in the areas of self-care, productivity, and leisure. Through a semi-structured interview, the COPM is a five-step process that measures client-identified problem areas in daily function. The importance of cooking to the research participants was rated on a scale of 1 to 10 through semi-structured interviews using the COPM procedure. In addition, the frequency of cooking was asked to confirm whether it was habitual or not.

NIRS recording and analysis

The procedure of this study is based on the report of Yücel et al. and Dans et al. [15,16]. NIRS was performed using a Spectratech OEG-16 (Spectratech Inc, Kanagawa, Japan). The OEG-16 has six light-emitting probes and six detection probes spaced 3 cm apart, allowing measurements on a total of 16 channels. Using two wavelengths of near-infrared light (770 and 840 nm), OEG-16 can measure changes in the concentrations of oxygenated hemoglobin (oxy-Hb) and deoxygenated hemoglobin in the blood.

**Figure 1:**
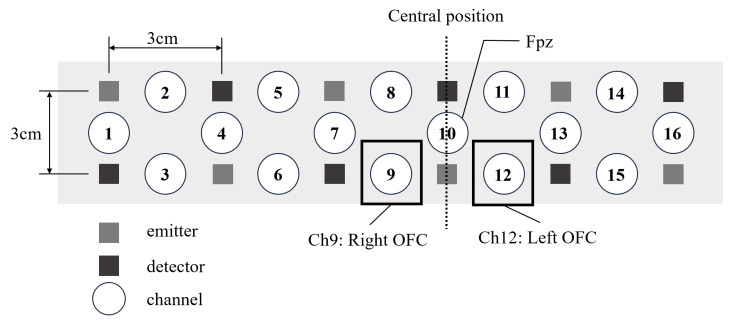
Near-infrared spectroscopy (NIRS) settings. OFC: Orbitofrontal cortex

The measurement task involved watching a cooking video, taking into account the influence of skin blood flow due to movement, as noted in previous studies [17]. The regions of interest were set to the right and left OFC because the OFC constitutes the reward system network. Virtual registration was used to identify the channel position and the location of the brain region [18]. In this method, the position of the virtual probe holder is transformed into a standard brain coordinate system (Montreal Neurological Institute) and projected onto the brain surface to estimate the brain region measured by the channel. The estimation error was within 13 mm, which is a practically acceptable level of spatial estimation accuracy. Each channel was arranged using the OFC (Ch9 and Ch12) and two of the 16 channels (Figure 1). The estimation probability of each channel was set based on the channel placement concerning the brain regions identified by Tsuzuki et al. [18]. The probe was mounted with its center aligned with Ch10 to match the estimated position of each channel.

The cerebral blood flow measured by NIRS includes components that are caused by intellectual activity when performing each task and components caused by vision when simply watching a video. This study aimed to extract the components caused by intellectual activity; therefore, it was necessary to remove the components caused by vision. To achieve this, we created two tasks, a target task and a control task. In the target task, the participants were required to engage in intellectual activity by watching a cooking video. In the control task, participants were required to perform an operation that did not require intellectual ability by simply looking at a still image. Next, we extracted the components caused by intellectual activity by subtracting the brain activation during the control task from the brain activation during the target task.

For NIRS data analysis, baseline fitting was conducted for 10 s before the task began, followed by a baseline correction process. A moving average of 5 s was applied to eliminate high-frequency components, and the average across three tasks was computed to determine the change in oxy-Hb. During baseline fitting, participants were presented with a white sheet of paper with a cross drawn on it and instructed to focus on the cross without engaging in any specific thoughts. The NIRS data were analyzed using Spectratech OEG16.

**Figure 2:**
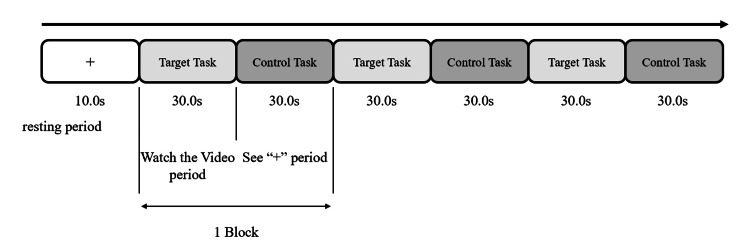
Procedure of block design.

The measurement task used a block design in which the target task consisted of watching cooking videos, and the control task consisted of looking at the “+” symbol written on a blank sheet of paper (Figure 2). In addition, we asked participants about their impressions of the videos after NIRS measurements.

Statistical analysis

For the statistical analysis, we classified COPM scores of ≥8 as “meaningful occupation execution group” (execution group) and ≤7 as “meaningful occupation non-execution group” (non-execution group) based on Miyata et al. [19] and compared the changes in oxy-Hb concentration between the two groups using the Mann-Whitney U test (significance level of 5%). The report by Miyata et al. used the Aid for Decision-making in Occupation Choice (ADOC) scale, which rates the importance of occupation on a five-point scale, with a score of 4 or more considered important. In this study, the COPM was used, so a score of 8 or more out of 10 was considered important. In addition, this study defined meaningful occupation as "occupation that is perceived to be important," so participants who scored 8 or more were classified as performing meaningful occupation.

## Results

**Figure 3:**
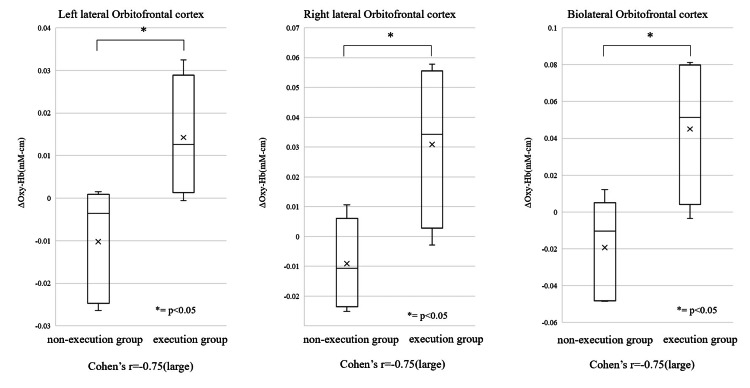
The results of the Mann–Whitney U test.

Four participants were assigned to the execution group (frequency of execution: various times a week for all participants) and four participants were assigned to the non-execution group (frequency of execution: various times a week for one participant, various times a month for one participant, and various times a year for two participants). The results of the Mann-Whitney U test (Figure 3) revealed significant differences in the left OFC; a significant difference was observed in the execution group 0.038 mmM·mm and the non-execution group -0.015 mmM·mm. In the bilateral OFC, a significant difference was observed in the execution group 0.058 mmM·mm and the non-execution group -0.042 mmM·mm (p<0.05). After watching the cooking videos, the execution group commented that the procedures and processes were helpful and expressed a desire to use them. In contrast, the non-execution group commented that they would enjoy cooking if they were able to cook well.

## Discussion

Meaningful occupations are hypothesized to be key in the healing process because they activate dopaminergic neural pathways in the brain. Studies using magnetic resonance imaging (MRI) have previously demonstrated that watching videos of significant work measured using the COPM test did not activate the reward system [17]. However, the most striking or self-selected changes activated dopaminergic neural pathways. Even so, there were differences in the categories of occupations perceived by study participants: a) as meaningful, b) as psychologically rewarding (likely to activate dopaminergic or reward neural pathways), and finally c) as meaningful and psychologically rewarding.

In this study, a significant difference was observed in the changes in oxy-Hb concentrations in the left and bilateral OFC in the execution group. The OFC reflects relative preferences for rewards [20], and motivation is formed based on these judgments. Comments made during the viewing suggest that the execution group perceived the video as valuable information useful when cooking. In the execution group of this study, cooking was perceived as an occupation that was both psychologically rewarding and meaningful, which may have led to motivation for subsequent occupation, which in turn may have influenced the increase in oxyhemoglobin concentration during video viewing. In occupational therapy practice, it is considered important that patients have a sense of purpose and that completing a task leads to confidence in their next action [21]. In other words, it is thought that the practice of meaningful occupations can activate the reward network centered on the OFC and that increased motivation can lead to the performance of further occupations, which in turn can contribute to the promotion of health and well-being.

Occupational therapists are healthcare professionals who focus on promoting health and well-being through occupation, placing the client at the center of their practice. Occupational therapy, particularly occupation-focused practice, aims to improve an individual’s physical and mental functions and daily living skills, thereby promoting health and well-being. Previous reports on occupational therapy concluded that engaging in meaningful occupations is directly linked to improvements in health and well-being [22-24]. Additionally, our results show that occupational therapy activates reward-related networks and increases motivation. This suggests that the practice of occupations that are meaningful to individuals may help them build their identity and achieve health and well-being.

In this study, we defined the act of cooking as a meaningful occupation depending on whether it is perceived as important or not. However, even when cooking is perceived as important, there are cases where it is done as an occupation that one likes to do or as an occupation that one has to do due to living alone or other circumstances. Therefore, in order to understand the meaning of occupation more broadly, it is necessary to consider and test how perceiving cooking as important affects an individual's perception of meaning, in addition to perceiving cooking as important.

Our study has certain limitations. In addition to the small number of participants in this study, the NIRS measurements were performed in a laboratory, and video was used to account for the effect on cerebral blood flow. Therefore, it is possible that the results differ from changes in cerebral blood flow during actual task performance. Future studies should perform measurements in the context of situations that resemble real-life environments and employ questionnaires in conjunction with the measurements.

## Conclusions

In this study, we measured changes in cerebral blood flow in the OFC while watching a cooking video for the execution group, who perceive cooking as a meaningful occupation that they consider important, and for the non-execution group, who do not. The results showed an increase in cerebral blood flow in the OFC in the execution group, which activated the OFC. The results suggest that it is important to focus on meaningful occupations that are important to the individual in order to activate the OFC and increase motivation. In other words, meaningful occupations can activate the reward system.
